# Vaccination and Timing Influence SIV Immune Escape Viral Dynamics *In Vivo*

**DOI:** 10.1371/journal.ppat.0040012

**Published:** 2008-01-25

**Authors:** Liyen Loh, Janka Petravic, C. Jane Batten, Miles P Davenport, Stephen J Kent

**Affiliations:** 1 Department of Microbiology and Immunology, University of Melbourne, Melbourne, Victoria, Australia; 2 Centre for Vascular Research, University of New South Wales, Sydney, New South Wales, Australia; National Institutes of Health–NIAID, United States of America

## Abstract

CD8+ cytotoxic T lymphocytes (CTL) can be effective at controlling HIV-1 in humans and SIV in macaques, but their utility is partly offset by mutational escape. The kinetics of CTL escape and reversion of escape mutant viruses upon transmission to MHC-mismatched hosts can help us understand CTL-mediated viral control and the fitness cost extracted by immune escape mutation. Traditional methods for following CTL escape and reversion are, however, insensitive to minor viral quasispecies. We developed sensitive quantitative real-time PCR assays to track the viral load of SIV Gag_164–172_ KP9 wild-type (WT) and escape mutant (EM) variants in pigtail macaques. Rapid outgrowth of EM virus occurs during the first few weeks of infection. However, the rate of escape plateaued soon after, revealing a prolonged persistence of WT viremia not detectable by standard cloning and sequencing methods. The rate of escape of KP9 correlated with levels of vaccine-primed KP9-specific CD8+ T cells present at that time. Similarly, when non-KP9 responder (lacking the restricting Mane-A*10 allele) macaques were infected with SHIV_mn229_ stock containing a mixture of EM and WT virus, rapid reversion to WT was observed over the first 2 weeks following infection. However, the rate of reversion to WT slowed dramatically over the first month of infection. The serial quantitation of escape mutant viruses evolving during SIV infection shows that rapid dynamics of immune escape and reversion can be observed in early infection, particularly when CD8 T cells are primed by vaccination. However, these early rapid rates of escape and reversion are transient and followed by a significant slowing in these rates later during infection, highlighting that the rate of escape is significantly influenced by the timing of its occurrence.

## Author Summary

Immune escape from AIDS virus–specific cellular immunity is common. The driving forces behind how quickly cellular immunity forces escape are poorly understood. We developed a novel assay for a common immune escape variant of SIV in macaques. This allowed us to sensitively track the rates of immune escape even when levels of escape mutant or wild-type virus were low. We found that prior immunization of macaques resulted in very rapid immune escape during acute infection. However, when escape starts to occur later, during chronic infection, the rate of immune escape is much more gradual. Thus, both prior vaccination and timing influence the rates of immune escape and provide a fuller picture of the effectiveness of T cell immunity to HIV.

## Introduction

CD8+ cytotoxic T lymphocyte (CTL) responses during acute HIV-1 and simian immunodeficiency virus (SIV) infection in humans and macaques, respectively, correlate with effective control of acute viremia [1–4]. CTL responses to both HIV-1 and SIV are, however, partially undermined by the evolution of viral escape [5–10]. Many CTL escape mutations evolve to select a single common escape motif, including the Mane-A*10-restricted KP9 SIV Gag epitope in pigtail macaques, which selects the K165R mutation to escape this response [11]. Our studies to date have suggested that CTL escape at KP9 can be rapid during acute infection, and completed within 1–2 weeks [11].

Reversion of CTL EM variants upon transmission to MHC-mismatched hosts have also been widely documented in both human and macaque settings [12–14]. These studies imply a significant fitness cost to the evolution of some Gag CTL escape mutations. Rapid reversion of the K165R escape mutant (EM) KP9 virus is observed in Mane-A*10 negative pigtail macaques infected with EM challenge stock SHIV_mn229_, suggesting a significant fitness cost of the K165R mutation [11].

To further elucidate CTL escape and reversion kinetics, we recently studied the *in vivo* effectiveness of different SIV Gag–restricted T cell responses in pigtail macaques [15]. Variable rates of escape and reversion occur across different Gag epitopes, implying that CTL effectiveness is also variable. These studies, like many others, used the traditional approach of cloning and sequencing a limited number of individual viruses (generally 10–20) to derive crude escape and reversion kinetics. Although widely accepted, this method is labour intensive and insensitive to the detection of subdominant viral quasispecies. For example, even where the viral load of an escape mutant virus is high (e.g. 10^5^ copies/ml), if the wild-type (WT) virus is 10-fold higher (e.g. 10^6^ copies/ml), the EM virus is difficult to either detect or accurately quantitate by cloning and sequencing unless many clones are sequenced. It is thus very difficult to track CTL escape and reversion beyond the initial selection phase without more sensitive technologies.

Quantitative real-time PCR (qRT-PCR) assays to track CTL escape mutations viral loads are needed to more accurately study the evolution of immune escape. Discrimination of WT from EM virus where a single base pair is altered would prove difficult using standard sequence-specific primers in PCR since WT primers may cross-react with the EM target with reasonable efficiency. However, a number of recent technical advances in sequence detection chemistries make this a more feasible task. Probes conjugated to a DNA Minor groove binding (MGB) moieties improve increase the stability and sensitivity for complementary sequence [16]. Similarly, Locked Nuclei Acid (LNA) LNA base modifications can be added to probes and primers. Locked nucleic acids are DNA analogs with a C4′-O2-methlene bridge incorporation in the sugar of a nucleotide. This modification allows for greater stability and specificity for complementary sequence [17,18].

Assays utilising real-time PCR technology and DNA probes for sequence-specific detection and measurement of common single nucleotide drug resistance mutations in HIV-1 infection have been developed [19]. These assays detected minor but clinically important levels of drug resistant virus variants typically missed by sequencing technologies. Peyerl and colleagues designed a molecular beacon-based assay to quantify common quasispecies in the Mamu-A*01-restricted SIV Gag CM9 CTL epitope [20]. However, this assay had a limited detection level of 5 × 10^2^ copies/ml of EM virus in 10^4^ copies/ml of total virus. This level of sensitivity can be problematic for samples assayed during acute infection where total virus can be in excess of 10^6^ copies and the minor quasispecies may only be present at 10^2^-10^3^ copies.

We report here the development of qRT-PCR assays utilising MGB-DNA probe and LNA-modified primer technologies to elucidate the finer kinetics of CTL escape and reversion. These assays sensitively detect minor viral quasispecies and allow comparative quantification of WT and EM viruses. We used these assays to refine our understanding of the evolution of KP9 SIV Gag CTL escape mutant viruses in pigtail macaques.

## Methods

### Macaques, Vaccinations, and Viruses

Experiments on outbred pigtail macaques (Macaca nemestrina) were approved by the University of Melbourne and CSIRO livestock industries Animal Ethics Committees. Pigtail macaques were MHC typed by reference strand-mediated conformational analysis for the Mane-A*10 allele which present SIV Gag epitope KP9 [21,22].

CTL escape and reversion kinetics was studied in 12 pigtail macaques (8 Mane-A*10+, 4 Mane-A*10 negative) in previously reported studies. Six Mane-A*10+ macaques were participating in DNA prime/recombinant fowlpoxvirus (rFPV) boost vaccine studies involving a viral challenge with either R5-tropic SHIV_SF162P3_ (animals 4292, 4246 [11,23]) or X4-tropic SHIV_mn229_ (vaccinated animals 6276, 5614, unprimed animals 6167, 5712 [24,25]). Further analyses of CTL escape were conducted in 2 naive Mane-A*10+ pigtail macaque infected with R5-tropic SIV_mac251_ (animal 5175, 5284 [26]). Reversion of the KP9 EM stock SHIV_mn229_ was studied in 4 Mane-A*10 negative pigtail macaques (4194, 4301, 1.1705, H20 [27]). We had previously studied escape and reversion at the KP9 epitope by extracting SHIV RNA from plasma and amplifying and sequencing cDNAs as previously described [11].

### Development of qRT- PCR for EM and WT Virus at KP9 Epitope

To quantify and validate the SIV Gag KP9 qRT-PCR, RNA standards were first constructed from WT and EM virus sequence. In short, pDNA clones previously sequenced at the SIV Gag KP9 were selected for EM and WT RNA standards, after confirmation from re-sequencing of the specified region. Prior to transcription of *in vitro* RNA, pDNA was linearized with the restriction endonuclease *Spe*I (Promega). In vitro RNA was transcribed using the ribomax T7 promoter kit (Promega) according to manufacturer's instructions. Following transcription, RNA was purified with the QIA RNA MinElute Kit according to manufacturer's instructions (Qiagen). Purified RNA was electrophoresed on a 1.5% agarose gel to check for the presence of correct transcript. Purity and concentration of RNA was derived to calculate RNA total copy number from UV spectrophotometry absorbance readings at 260nm using Ultrospec 3100Pro UV/Vis spectrophotometer (Amersham Biosciences).

To quantify WT and EM viral loads, standard curves were produced by reverse transcribing 10^9^ copies of RNA standard and diluting cDNA 10-fold (10^7^-10^1^ copies) in nuclease-free water to produce WT and EM cDNA standards for analysis. RNA was extracted from serial plasma samples isolated from infected pigtail macaques using a QIAmp Viral RNA mini kit (Qiagen). 10μl of RNA was subjected to two-step qRT-PCR. The first step involved reverse transcription of RNA in a 30 μl reaction carried out as follows: 10μl of RNA was added to a PCR mix containing: 10× PCR Buffer II (ABI), 10× PCR Buffer A, 5mM of MgCl_2_ (Promega), 10μM of random hexamers, 0.5mM of each dNTP (Promega), 20U of RNasin (Promega), and 20U of SuperScript III RNase H- reverse transcriptase (Invitrogen). The cycling conditions were: 25°C for 15 min, 42°C for 40 min, and 75°C for 5 min, using GeneAmp 9700 PCR thermal cycler (ABI) or Reaplex^4^ (Eppendorf). 50μl qRT-PCR reactions were achieved by the addition of 30μl of cDNA to 20μl of qRT-PCR mix containing: 17μl of 2x TaqMan universal mastermix (ABI), 100nM of Minor Groove Binding (MGB)-Gag-KP9: 5′ 6FAM- CCTGGCACTACTTCT- MGBNFQ 3′ where NFQ = Non-fluorescent quencher (ABI), 400nM of reverse primer No. 179: 5′ CCTTCTGACAGTGCCTGAA 3′ for KP9, 400nM of WT forward primer or EM forward primer was also added to each PCR reaction. The WT primers for KP9 was No.172 5′ GG+GTAAAATT+GATA+GAGGAAAAGA+A 3′ (+ precedes LNA-modified bases; mutated codons are denoted by underlined bases). The forward primers for EM KP9 was No.174: 5′ GG+GTAAAATT+GATA+GAGGAAAAGA+G 3′. To validate the total virus (WT +EM KP9) quantified an additional SIV Gag forward primer #230 5′ GG+GTAAAATT+GATA+GAGGAAAAGA 3′ which amplifies both targets was combined with primer #179 and the MGB-probe to separately quantify total virus.

The cycling conditions were as follows: 50°C for 2 min, 95°C for 10 min, followed by 45 cycles of: 95°C for 15 sec, 63°C for 60 sec, using an ABI Prism 7700 sequence detection system PCR thermal cycler or an Eppendorf Realplex^4^ cycler. Analysis was performed using SDS applications version 1.9 (ABI) or Eppendorf Realplex^4^ software. Baselines were set 2 cycles earlier than real reported fluorescence and threshold value was determined by setting threshold bar within the linear data phase. Samples amplifying after 40 cycles were regarded as negative, and correspond to < 1.5-Log_10_ SHIV/SIV RNA copies/ml of plasma (threshold value of quantification).

### Characterization of Reversion and Escape Rates

We define reversion rate as the absolute difference in growth rates between the wild-type (W) and mutant (M) in a given time interval [11]. If *f_W_*(*t*) is the fraction of wild-type and *f_M_*(*t*) is the fraction of escape mutant clones at time *t*, we can calculate the time-dependent reversion rate *R* from the proportions of clones of each type at the end points *t_s_* and *t_e_* of the time interval,

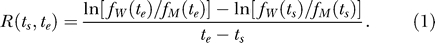



Negative reversion rate implies that the mutant has a growth advantage over wild-type.

Escape rate *E* is defined as the difference of growth rates of the escape mutant and wild-type,

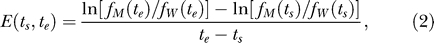
so that the negative escape rate implies that the fraction of wild-type increases in the given time interval. The expressions for reversion and escape rates assume exponential growth/decay of viral load within the time interval. The justification for this assumption is that, when viral load changes rapidly between two time points, its time dependence is better described by an exponential than by a linear function. When the change is slow, linear and exponential interpolations give almost the same results (not shown). Correlations between reversion / escape rate and other parameters were performed using non-parametric (Spearman) correlations.

## Results

### SIV Gag KP9 qRT-PCR Sensitively Quantitates Minor Viral Quasispecies

**Figure 1:**
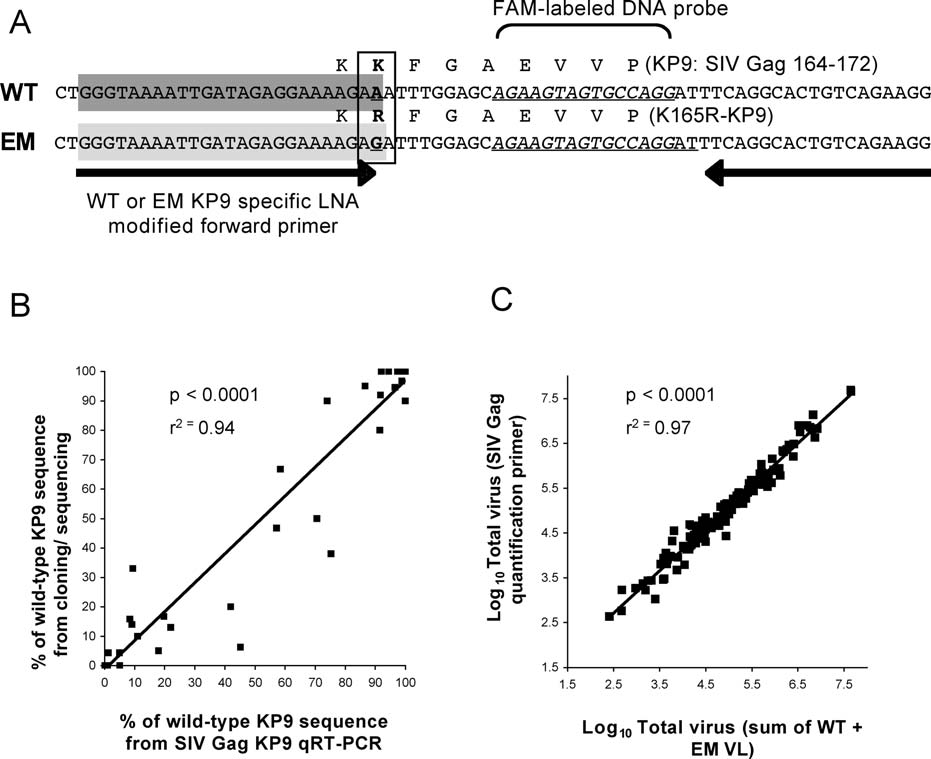
SIV Gag_164–172_ KP9 qRT-PCR Assay Schematic and Validation (A) Wild-type (WT)-specific locked nucleic acid (LNA) primer sequence highlighted in dark grey, escape mutant–specific sequence highlighted in light grey. Bold underlined nucleotides represent mutations within epitopes. DNA reverse primer and FAM-labeled minor groove binding-DNA probe allow quantification of both WT and EM target. (B) Validation of proportions of EM and WT virus at KP9 comparing qRT-PCR to cloning and sequencing on 46 plasma samples. (C) Validation of the sum of the EM and WT VL detected by qRT-PCR compared to total VL detected using a quantification primer outside the KP9 epitope on 129 plasma samples.

To more sensitively track CTL EM and WT viremia, we developed and analyzed a series of qRT-PCT assays on plasma viral RNA. We initially studied labelled Taqman probes specific for either EM or WT sequence as previously reported [20], but found the use of LNA-modified sequence-specific forward primers much more sensitive and specific (not shown). The combination of LNA-modified forward primers specific for WT or EM viruses at the KP9 Gag epitope with MGB-DNA probes to quantify either target was validated as an alternative method to labour intensive cloning and sequencing of viral populations (Figure 1A). The SIV Gag KP9 qRT-PCR assays were validated on WT and EM RNA standards created from *in vitro* transcription of plasmid DNA. Primers were validated for specificity by adding the WT forward primer to serial 10-fold dilutions of EM RNA standards; the inverse was performed with the EM primer. The KP9 forward primer was only very minimally cross-reactive in the presence of massively excess target (> 2 × 10^5^ copies) and even then the amplification of mismatched target was ≥10 cycles after that of matched target (Table 1).

**Table 1:**
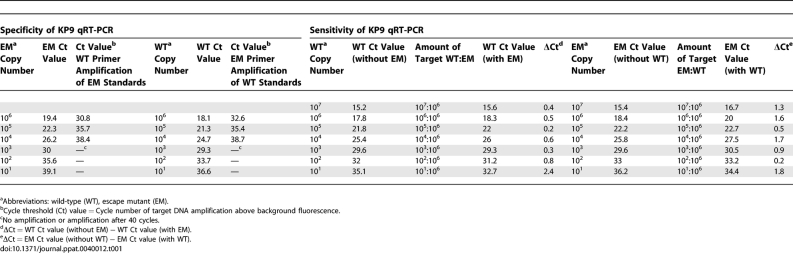
SIV Gag_164–172_ KP9 Quantitative Real-Time PCR Validation

To more rigorously examine the specificity and sensitivity of qRT-PCR, a series of mixed dilutions of WT and EM RNA standards were prepared to mimic the mixed target ratios that may occur during SIV/SHIV infection. The cycle threshold (Ct) value (cycle number at which amplification cross above background fluorescence) was then compared to amplification of WT target without competing target (EM) (Table 1). Similar results were observed with the EM forward primers. The KP9 qRT-PCR assays detected up to 10 copies of WT target in an excess of 10^6^ EM copies, although the Ct value for detection of mixed WT KP9 was 2.4 cycles earlier than detection of 10 copies of WT alone. To ensure that this was not due cross reactivity of the WT primer, the PCR product was bulk sequenced, and the chromatogram confirmed no mixed base at the position of the escape mutation. We then compared the proportions of KP9 WT and EM virus by qRT-PCR assay in a large series of pigtail macaque plasma samples (46 samples) that had previously undergone cloning and sequencing with varying levels of WT and EM clones. The assays were tightly correlated (r^2^ = 0.94, p <0.0001, Figure 1B). We further validated the assay by comparing the sum of the WT and EM VL with total VL detected using a quantification forward primer outside the K165R mutation on 129 plasma samples. The total viral loads detected by both methods were tightly correlated (Figure 1C).

### Kinetics of Escape Mutant Viremia

**Figure 2:**
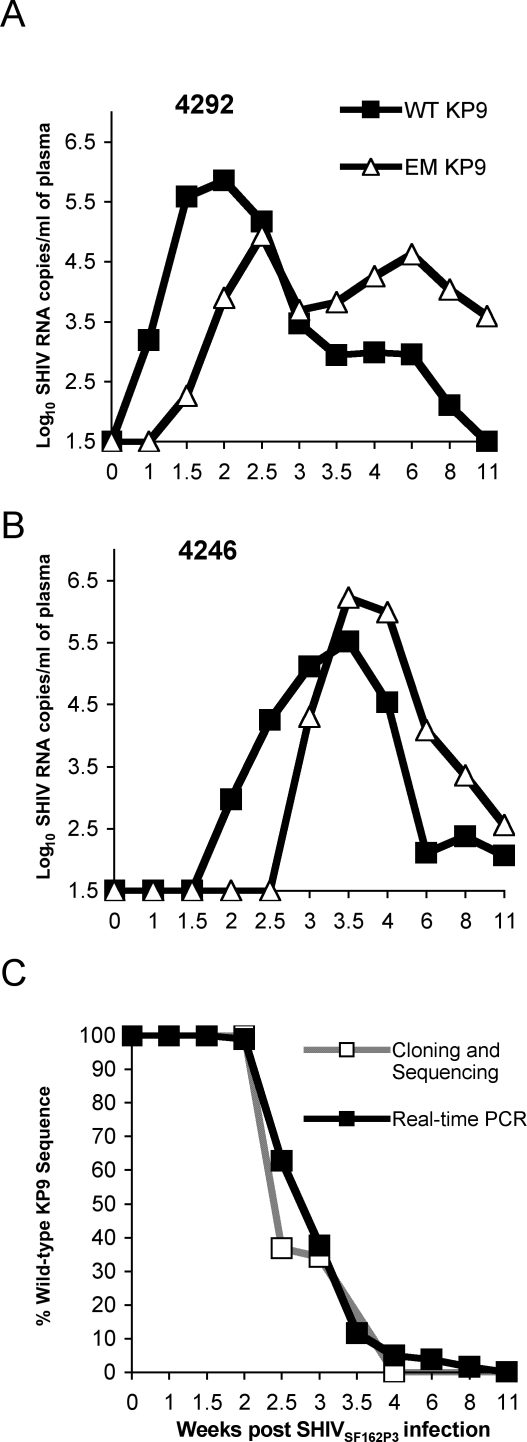
Minor Populations of Wild-Type KP9 Virus Can Be Detected by qRT-PCR during Chronic Infection SIV Gag KP9 qRT-PCR analysis was performed on serial RNA samples extracted from plasma from Mane-A*10 pigtail macaques (A) 4292 and (B) 4246 infected with SHIV_SF162P3_. Wild-type and escape mutant KP9 viruses are represented by closed squares and open triangles, respectively, throughout. Cloning and sequencing (open squares) was compared to qRT-PCR (closed squares) to establish the proportions of wild-type KP9 virus in animal 4292 (C). A mean of 25 clones were sequenced across KP9 for each time point.

We previously studied CTL escape at KP9 in DNA and rFPV-immunized Mane-A*10+ pigtail macaques infected with SHIV_SF162P3_, a challenge stock that contains WT SIV_mac239_ *gag* [11,23]. The kinetics of escape derived from cloning and sequencing of plasma viral cDNA indicated that rapid escape occurred during acute infection. To further define the fine kinetics of escape and validate the in vivo utility of our novel assay, we applied the KP9 qRT-PCR assay on a time course of RNA samples extracted from two Mane-A*10+ macaques. From qRT-PCR we can measure EM and WT viral loads comparatively. Wild-type virus decays rapidly between 2–3.5 weeks after infection in both animals (Figure 2A and 2B). The sensitivity of qRT-PCR is demonstrated in animal 4292, where minor amounts of EM virus starts to become detectable during the peak of viremia when WT virus is 2-Log_10_ greater than EM virus. These minor populations of EM virus are difficult to detect by cloning and sequencing (Figure 2A and 2C). Similarly, minor WT virus populations are detectable well after the peak of infection (week 4), at a time when escape appears “complete” by cloning and sequencing (Figure 2A and 2C). When plotted as a proportion of WT virus, *in vivo* kinetics of escape in acute infection are very similar between the qRT-PCR assay and cloning/sequencing technologies (Figure 2C). However, qRT-PCR allows estimation of escape rate over a much longer period post-infection. This shows a considerable slowing in the rate of escape after the first ∼4 weeks on infection, a phenomenon not measurable using standard cloning and sequencing approaches.

### Rates of Reversion of Escape Mutant Virus

**Figure 3:**
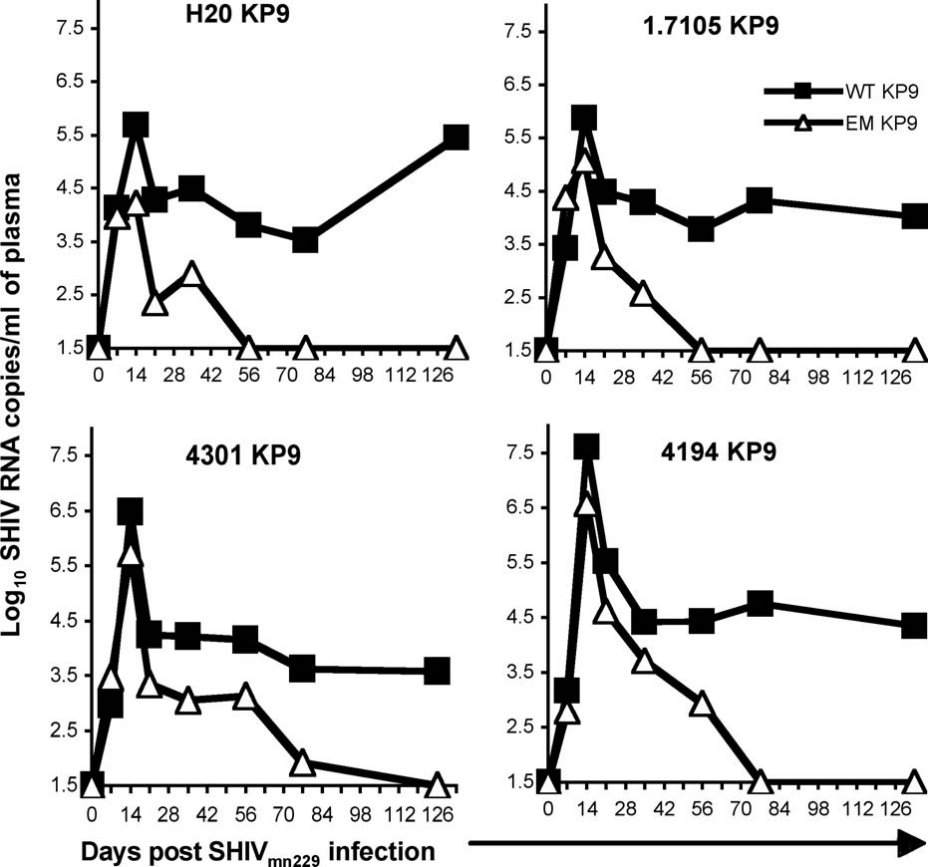
*In Vivo* Reversion of KP9 Escape Mutant Virus The kinetics of reversion at SIV Gag KP9 was assessed by qRT-PCR from serial RNA samples extracted from plasma in 4 Mane-A*10 negative pigtail macaques infected with the EM KP9 SHIV_mn229_ reversion (11.2% WT KP9 by qRT-PCR).

Escape mutant viruses usually incur some reduced level of replicative fitness as a result of the escape mutation (the “fitness cost of escape”); this is most clearly demonstrated when EM virus reverts to the fitter WT upon transmission to MHC-mismatched hosts [11,12,13,28]. We previously studied reversion of WT KP9 virus in Mane-A*10+ macaques challenged with the EM challenge virus SHIV_mn229_. This virus is derived from a SHIV_HXB2_ stock passaged through Mane-A*10+ pigtail macaques [11]. The SHIV_mn229_ stock was 90.9% (40/44 clones) K165R EM virus and 9.1% (4/44 clones) WT virus by cloning and sequencing. The challenge virus stock was 11.2% WT by our qRT-PCR. Reversion of the EM SHIV_mn229_ stock was rapid in all 4 Mane-A*10 negative animals studied. The decay of EM virus occurred after peak of viremia and the EM virus was present only at minor or undetectable levels by 8 weeks after infection (Figure 3).

**Figure 4:**
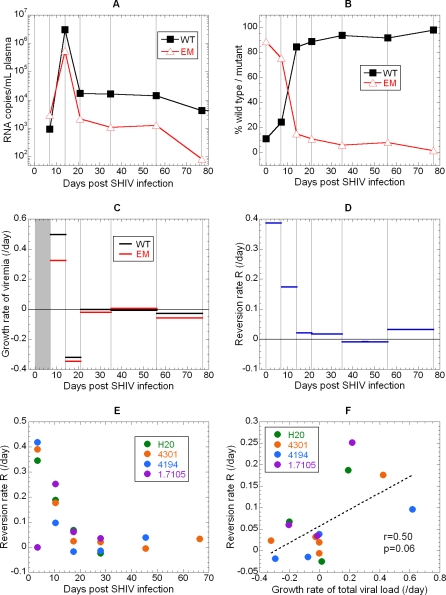
Rapid Early Reversion of KP9 from Escape Mutant to Wild-Type Virus The levels of WT and EM virus in a representative animal (#4301) are shown (A), as is the fraction of EM and WT virus over time (B). The average growth rates of WT (black) and EM (red) virus in different time intervals are calculated from the viral loads in (C), and the rate of reversion to WT is shown in (D). Growth rates could not be evaluated during the first week because the viral loads at day 0 were below detection (grey rectangle in [C]), but reversion rate was calculated from the known percentages of EM and WT in the inoculum. A comparison of the variation of reversion rate with time is shown for the different animals (E), and the correlation between reversion rate and total virus growth rate (F).

The rate of reversion to WT virus estimated using qRT-PCR was very similar to that observed using conventional cloning and sequencing during the first 2 weeks of infection (mean reversion rate of 0.38 for day for cloning and sequencing [11], and 0.35 per day for qRT-PCR). However, after this time the rate of reversion to WT slowed significantly (Figures 3 and 4), and significant levels of EM virus persisted for several weeks. It has previously been noted that the maximal reversion rate will only be observed when total viral load is in exponential growth, and that overall viral dynamics will determine the observed rate of reversion [11,29]. Consistent with this, the rate of reversion was positively correlated with the overall viral growth rate (Figure 4F, r = 0.50), approaching statistical significance (p = 0.06). This suggests that, like the rate of escape described above, the rate of reversion slows dramatically after the first few weeks of infection.

### Transient Reversions and Re-Escape Timing Is Influenced by Prior Vaccinations

Reversion and re-escape of EM virus in MHC-matched hosts has been correlated with the expansion of epitope-specific CTL [25,30]. Our group recently demonstrated that in animals responding to the KP9 epitope, infection with a mix of WT and EM virus results in an early phase of reversion to WT (presumably due to a delay in the induction of the CTL response [31]), followed by a later phase of immune escape due to CTL mediated killing of WT infected cells. The extent of this early reversion is inversely correlated with the level of vaccine-induced KP9-specific CTLs in EM KP9 SHIV_mn229_-infected Mane-A*10+ pigtail macaques [25]. However, the timing and kinetics of reversion and re-escape were assessed only at a few time points by insensitive cloning and sequencing techniques. We hypothesised that the very limited dynamic range of cloning and sequencing probably underestimates the degree to which WT virus is controlled by prior vaccination.

**Figure 5:**
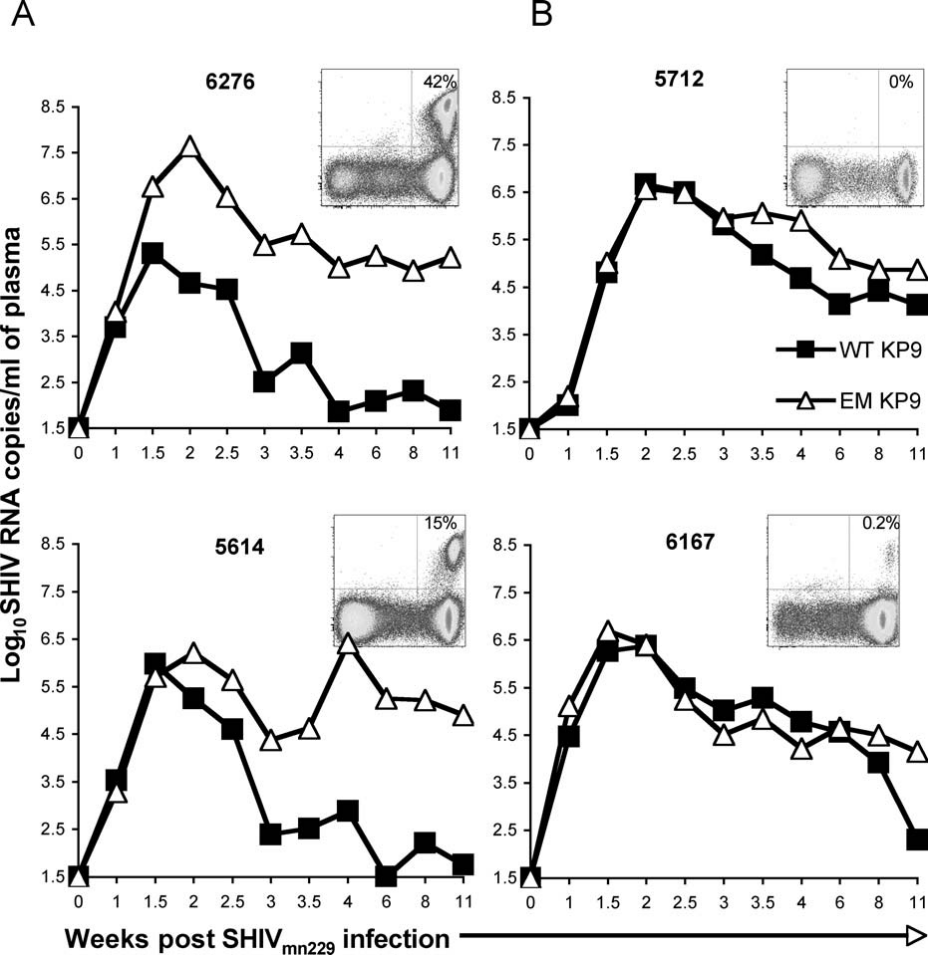
Transient Reversions and Re-Escape Timing Is Influenced by Prior Vaccination and Levels of KP9-Specific CTL Wild-type (closed squares) and escape mutant (open triangles) KP9 was measured by SIV Gag KP9 qRT-PCR to compare the kinetics of transient reversion and re-escape in four Mane-A*10+ pigtail macaques; 6276 and 5614 were vaccinated with DNA/FPV prime/boost vaccines (A), 5712 and 6167 were the control animals and had no prior induction of KP9-specific T cells (B). Levels of KP9-specific CD8 T cells detected by the Mane-A*10/KP9 tetramer at 2 weeks after challenge for each animal are shown as an inset. The *y*-axis is the KP9 tetramer and the *x*-axis is CD8. Cells shown are gated CD3+ lymphocytes.

SIV Gag KP9 qRT-PCR analysis of two vaccinated and two control animals without prior induction of KP9-specific T cells yielded contrasting results (Figure 5). Animals 6276 and 5614 were vaccinated with DNA/FPV prime/boost vaccines and DNA vaccination only, respectively, and had detectable levels of KP9-specific CD8 T cells at infection (0.06% and 0.04% of CD8 T cells, respectively, by KP9/MHC-tetramer) that expanded to high levels after challenge (peaking at up to 42% 2 weeks after challenge, Figure 5A [25]). Both WT and EM KP9 virus expanded very early after SHIV_mn229_ inoculation up to the peak of viremia (week 2 post-infection); however, this is followed by an abrupt loss of WT virus to low levels by week 3 where EM virus dominates as KP9-specific T cells expand. Furthermore, the striking difference in absolute quantities of EM and WT are 2.5-Log_10_ or greater week 3 post-infection, and this minor population of WT virus is unlikely to be detectable by cloning and sequencing.

The situation is very different in un-primed animals 5712 and 6167. These animals had 0.00% KP9-specific T cells at the time of challenge, and only up to 0.2% by 2 weeks after challenge (Figure 5B). The WT virus decay is gradual, and this is most clearly observed at week 3 where in un-primed animals WT and EM virus are co-dominant, and the viral load is ∼5 Log_10_ (Figure 5B). These findings illustrate the extent to which prior vaccination impacts the rates of killing of WT viremia.

**Figure 6:**
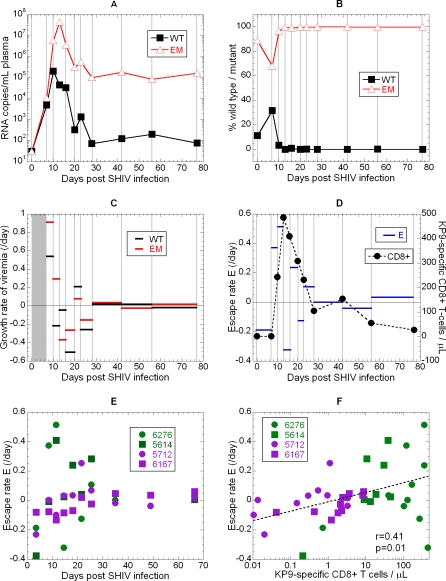
Early Reversion and Re-Escape Rates at the KP9 Epitope The levels of KP9 wild-type and escape mutant in a representative animal (#6276) following infection with the SHIV_mn229_ virus (containing 11.2% WT and 88.8% EM virus) (A). The fraction (B) and growth rates (C) of WT and EM virus over time are also shown. The rate of escape (D) is negative immediately after infection (as the WT virus takes over, prior to CTL effects) Growth rates could not be evaluated during the first week because the viral loads at day 0 were below detection (grey rectangle in [C]), but we calculated the difference in growth rates rate from the known percentages in the inoculum. (E) The variation of escape rate with time is shown for the different animals that were either vaccinated (green symbols) or unvaccinated (purple symbols). (F) The rate of escape is significantly correlated with the number of KP9 responding CTLs detected by the KP9/Mane-A*10 tetramer.

The rates of both reversion and escape are fastest during early infection, and then slow by 5 weeks post-infection. In the vaccinated animals, an initial rapid burst of escape was observed around day 10–20, followed by a significant slowing in escape. This paralleled the levels of KP9-specific CTLs (Figure 6A–6E). WT virus was still detectable out to week 11 after infection at low levels (∼1,000-fold lower than EM). By contrast, in the animals without prior induction of KP9-specific T cells by vaccination, escape did not occur until later (day 20 onwards). When escape did occur it was at a slower rate, similar to the rate observed in the vaccinated animals at these later times.

The pattern of rapid early escape followed by a significant slowing is consistent with the rapid early reversion seen above and suggests again an association with overall viral dynamics, with rapid escape at periods of high overall viral growth and decay. However, the rate of escape is also thought to be determined largely by the magnitude of the CD8+ T cell pressure on the virus (which also varies during acute infection (Figure 6C)). Consistent with this, the rate of escape was significantly correlated with KP9-specific CD8+ T cell levels (r = 0.41, p = 0.01, Figure 6F).

### Slow Escape during Chronic SIV Infection

**Figure 7:**
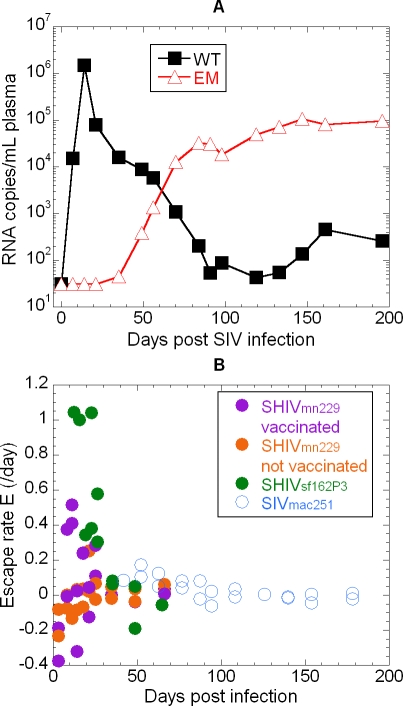
Slow Escape during Chronic SIV Infection and Comparison of Escape Rates across Multiple Viruses (A) KP9 EM and WT viremia was studied in a naïve pigtail macaque (#5175) infected with SIVmac251. (B) The rate of escape over time at the KP9 epitope following infection with three different strains of virus: In SHIV_mn229_ (orange circles are not vaccinated, purple circles are vaccinated), early reversion is followed by viral escape. In SHIV_SF162P3_ infection (green circles), escape occurs early and the escape rate is fast. In SIV_mac251_ infection (open blue circles), escape occurs late and the escape rate is slow.

Our initial studies demonstrated a very rapid rate of escape at the KP9 SIV Gag CTL epitope during acute infection, followed by a slower rate of escape. We hypothesised that when escape begins later during chronic infection, it may have slower kinetics since the overall viral expansion rates are negligible at this time. To further study the concept of slow escape during late infection, we analysed serial plasma samples from naïve Mane-A*10+ macaques infected with WT SIV_mac251_ that we had previously noticed appeared to have slower kinetics of escape by a limited amount of cloning and sequencing [26]. The qRT-PCR assay confirmed a slow replacement of WT with EM virus over 8 weeks (weeks 5–13 after infection, Figure 7A). This is consistent with stable overall viral loads during this time and low rates of escape (Figure 7B, open blue circles)

## Discussion

CTL responses drive mutational escape in both HIV-1 and SIV infection [5,7,8,9,10,11,32]. Previous analyses of CTL escape and reversion of EM viruses relies on cloning and sequencing limited numbers of cDNA clones; this method is expensive, labour intensive and also insensitive to viral quasispecies of low frequency. Higher-throughput assays with larger dynamic ranges are required to more accurately monitor later phases of mutational escape and reversion. We show that sensitive qRT-PCR assays can be developed to quantify minor viral quasispecies utilising MGB-DNA probe and LNA-modified primer technologies. The qRT-PCRs can detect greater than a 10^4^ virus copy difference between WT and EM viruses, which was validated both *in vitro* and *in vivo*. The dynamic range of discrimination of this assay surpassed our own and other [20] attempts using molecular beacon technologies.

We studied the longitudinal kinetics of escape in individual animals by measuring the viral load of EM and WT virus at KP9 over time. The rate of reversion of EM to WT virus was rapid early, followed by a marked slowing in reversion rate, consistent with previous predictions that the maximum reversion would only be observed during exponential viral growth. This study only monitored reversion in circumstances where both WT and EM were present in the initial inoculum. However, it suggests that where WT virus is not present in the inoculum and arises instead through mutation of EM virus later during the course of infection, we would expect to observe slower rates of reversion even for the same overall difference in viral fitness.

Similarly, we studied the rate of escape from immune pressure in three circumstances (Figure 7B): (i) where EM arose due to mutation of WT virus during acute infection with SHIV_SF162P3_, (ii) where both EM and WT were present in the initial inoculum (with SHIV_mn229_) and early reversion to WT was followed by escape, and (iii) where EM arose due to mutation of WT late during infection with SIV_mac251_. Although different parental viral strains were used in these studies, comparison of the rates of escape across the models suggests a similar pattern to that observed in reversion: significantly higher rates of escape in early infection, which tended to stabilise later. Notably, where escape only occurred late (in the unvaccinated SIV_mac251_ and SHIV_mn229_ infected animals), the rate of escape was similar to the rate observed in the vaccinated animals at the same time (even though the vaccinated animals had earlier shown a burst of rapid early escape). It is difficult to determine in this case whether the slower rates of escape later in infection were determined by viral dynamics or KP9-specific CD8+ T cell dynamics, since the number of KP9-specific cells followed a similar trend to the escape rate, and the rate of escape was significantly correlated with KP9-specific CTL numbers (Figure 6F). Another possibility is that CTLs lose some functionality during chronic infection, resulting in less selective pressure during escape. However, it appears that vaccination was associated with earlier and more rapid escape, and ultimately also WT virus was suppressed to lower levels compared to EM.

Effective vaccination clearly modulates the evolution of mutational escape and reversion. We recently showed that vaccination induces KP9-specific T cells that control reversion of EM viruses in MHC-matched pigtail macaques [25]. In this study, where WT and EM KP9 viremia can be measured compared by sensitive qRT-PCR, the effect of prior vaccinations is emphatically demonstrated. A marked 3-Log_10_ loss in WT viral load occurs within 3 weeks of infection in vaccinated animals; vaccination is effectively controlling the WT virus (but not, of course, the EM virus).

A number of recent studies have measured the rate of selection of WT and EM virus as a way of estimating the strength of immune pressure and/or fitness cost of escape mutation [11,25,33]. However, previous studies, including our own, have not taken into account the effects of the time when escape is first detected on the observed rate of escape. For example, in our previous study we observed the rate of selection of EM virus in SHIV_mn229_ and SHIV_sf162_ infected animals, where escape occurred in the first few weeks of infection (Figure 7B) [11,25]. As discussed above, the rate of escape was high in the first few weeks of infection, but rapidly slowed by 1–2 months after infection. By contrast, during SIV_mac251_ infection, escape was not observed until later in infection, starting after the first month. A direct comparison of early escape rates observed in SHIV and SIV_mac251_ infection would suggest that escape is slower in SIV_mac251_ infection, and thus that immune selection may be weaker. However, if one takes into account the timing of escape, the rate of selection during the second month of infection was remarkably similar between SHIV and SIV_mac251_ infections (compare open blue circles with green / orange circles), and in fact escape may even have been faster in SIV_mac251_ infection. Thus, the assumed low escape rate (and presumed low selection pressure) in SIV_mac251_ was most likely a factor of the timing of escape, not indicative of the potential effectiveness of vaccine-induced responses in controlling the virus through this epitope.

Our findings have important implications when trying to infer immune selection pressure from escape rates. For example, it has been suggested that the CTL response in humans is less effective than in macaques, because of the faster escape rates observed in macaques [33]. However, escape is invariably observed later in infection in human studies, due to the unknown date on infection. Even when the analysis is limited to “acutely infected” humans, this rarely includes cases where escape is observed around the peak of viraemia. Thus, given the link between the timing and rate of escape, it is not surprising that escape appears slower in humans than macaques, as when escape is observed it is usually substantially later in infection. Our studies predict that if samples can be obtained from subjects with acute HIV infection where escape or reversion begins to occur early, that the rates of escape and reversion observed will be rapid (and potentially completely missed if samples are only studied in chronic infection).

Understanding when and at what rate escape occurs at different epitopes is complex. Escape may occur late because the mutations required are rare, or because the EM virus is only weakly selected once it occurs. The strength of immune control and the fitness costs of escape mutation have been suggested as criteria for selection of optimal vaccine epitopes [34]. However, the present study suggests a note of caution in interpreting and comparing escape rates between different infections [33].

Although we describe in this report novel sensitive qRT-PCR assays to detect minor viral quasispecies, several limitations of this assay should be noted. First, although our assay can specifically discriminate WT from EM viruses, only the EM virus that the assay was designed for can be distinguished, and other mutants that may arise will not be detected, as it is the LNA-Forward primer that drives the specificity of this qRT-PCR. Additional qRT-PCRs could be designed for rarer escape mutations if needed. For example, we have rarely detected a typically transient mutation with KP9 at position 9 (P172S) during the evolution of the canonical K165R escape [11,26]; understanding the relationship between the 2 mutations could be obtained with additional qRT-PCR assays. With regard to the sensitivity of this assay, although the greater dynamic range allows for detection of minor quasispecies, the integrity of the plasma/RNA sample is critical for accurate quantification of the sample—the plasma/RNA must be stored appropriately with minimal freeze/thaws to avoid RNA degradation. The sensitivity of this discriminatory viral load assay could be further improved by pelleting virions using ultracentrifugation of larger plasma samples [35].

In summary, we have developed qRT-PCRs to measure the kinetics of CTL escape and reversion. We show that both the timing of escape and reversion and prior vaccination influence the dynamics of EM viremia. These findings facilitate a better understanding of the evolution and fitness of escape mutant viruses occurring during HIV-1 and SIV infection.
